# Loop-armed DNA tetrahedron nanoparticles for delivering antisense oligos into bacteria

**DOI:** 10.1186/s12951-020-00667-6

**Published:** 2020-08-04

**Authors:** Yue Hu, Zhou Chen, Xinggang Mao, Mingkai Li, Zheng Hou, Jingru Meng, Xiaoxing Luo, Xiaoyan Xue

**Affiliations:** 1 Department of Pharmacology, Fourth Military Medical University, No. 169, Changle West Road, Xi’an, 710032 Shaanxi People’s Republic of China; 2 Department of Neurosurgery, Xijing Hospital, Fourth Military Medical University, Xi’an, China

**Keywords:** DNA tetrahedron, Antisense antibiotics, Nanoparticles, Drug delivery system

## Abstract

**Background:**

Antisense oligonucleotides (ASOs) based technology is considered a potential strategy against antibiotic-resistant bacteria; however, a major obstacle to the application of ASOs is how to deliver them into bacteria effectively. DNA tetrahedra (Td) is an emerging carrier for delivering ASOs into eukaryotes, but there is limited information about Td used for bacteria. In this research, we investigated the uptake features of Td and the impact of linkage modes between ASOs and Td on gene-inhibition efficiency in bacteria.

**Results:**

Td was more likely to adhere to bacterial membranes, with moderate ability to penetrate into the bacteria. Strikingly, Td could penetrate into bacteria more effectively with the help of Lipofectamine 2000 (LP2000) at a 0.125 μL/μg ratio to Td, but the same concentration of LP2000 had no apparent effect on linear DNA. Furthermore, linkage modes between ASOs and Td influenced gene-knockdown efficiency. Looped structure of ASOs linked to one side of the Td exhibited better gene-knockdown efficiency than the overhung structure.

**Conclusions:**

This study established an effective antisense delivery system based on loop-armed Td, which opens opportunities for developing antisense antibiotics.
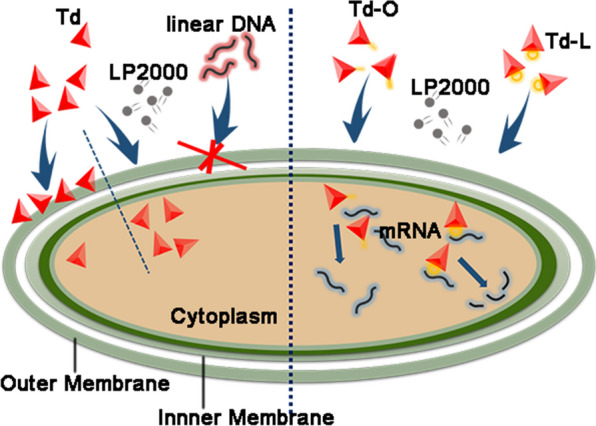

## Background

Infections caused by antibiotic-resistant bacteria have raised public concerns worldwide. Among the assorted solutions, such as antibiotic combination [1] and small-molecular compound screening [2], the antisense antibacterial strategy has drawn considerable attention due to its convenient target selection, safety and decreased likelihood of inducing antibiotics resistance [3]. By specifically blocking the expression of targeted genes in bacteria, antisense oligonucleotides (ASOs) have shown great potential to kill bacteria [4] or reverse the resistance of bacteria [5]. However, without vectors, ASOs can hardly enter bacterial cells because of their high molecular weight [6] and the complex structure of the bacterial cell wall [7], which are the main obstacles for ASOs in therapeutic field. Thus far, a multitude of materials have been studied for ASOs delivery, including cell-penetrating peptides (CPPs) [4], vitamin B_12_ [8], liposomes [9] and other polymers [10]. Among these materials, CPPs are the most widely used and have proven to be effective when covalently linked with ASOs, but their utilization is impeded by their cytotoxicity at high concentrations and potential immunogenicity to the host [11]. Therefore, the development of alternative carriers is essential.

Recently, DNA nanomaterials have offered entirely new avenues for drug delivery systems [12]. Based on Watson–Crick base pairing, DNA nanoparticles exhibit excellent advantages over traditional nanoparticles, including precise manipulation of shape and size, biocompatibility, nontoxicity and increased likelihood of intelligent modification [13]. Particularly, DNA tetrahedra (Td) is widely used due to its simple preparation, rigid structure and flexible optimization [14]. In previous studies, it has been verified that Td can enter live HEK cells without transfection agents [15] and deliver small-molecule compounds [16] or nucleic acid drugs [17, 18] into eukaryotic cells. Furthermore, Td has great flexibility for structural modification by aptamers [19], folate acids [17] or tumor-penetrating peptides [20], exhibiting considerable potential for facilitating versatile drug delivery.

Nevertheless, only a few studies have focused on the application of Td as a delivery system for antibacterial agents. Leong reported that Td intercalated with actinomycin D could be internalized efficiently by *Escherichia coli* (*E. coli*) and *Staphylococcus aureus* (*S. aureus*) and showed stronger antibiotic effects than free actinomycin D in vitro [21]. Other studies demonstrated that Td incorporated with peptide nucleic acids targeting *bla*_CTX-M-group 1_ in cefotaxime-resistant *E. coli* [22] or *ftsZ* in methicillin-resistant *Staphylococcus aureus* (MRSA) [23] could enter bacteria to restore sensitivity to cefotaxime or to inhibit bacterial growth by inhibiting targeted genes. These results imply that Td could be a carrier to deliver ASOs into bacteria. However, the delivery efficiency of Td in different strains and the factors that influence Td into bacteria, as well as the type of Td structure or linkage modes with ASOs remain unclear.

In this study, we investigated the uptake characteristics and efficiency of Td by different bacterial strains, including *S. aureus*, *E. coli*, *Shigella flexneri* (*S. flexneri*), *NDM1*-*Klebsiella pneumoniae* (*K. pneumoniae*), *multiple*-*drug resistant Pseudomonas aeruginosa* (*P. aeruginosa*) and *Acinetobacter baumannii* (*A. baumannii*). Next, we designed two types of linkages modes between Td and ASOs targeting *gfp*, encoding green fluorescent protein (GFP), or *acpP*, encoding the acyl carrier protein (Acp), and assessed the efficiency of delivery and gene knockdown in *E. coli*.

## Results and discussion

**Fig. 1:**
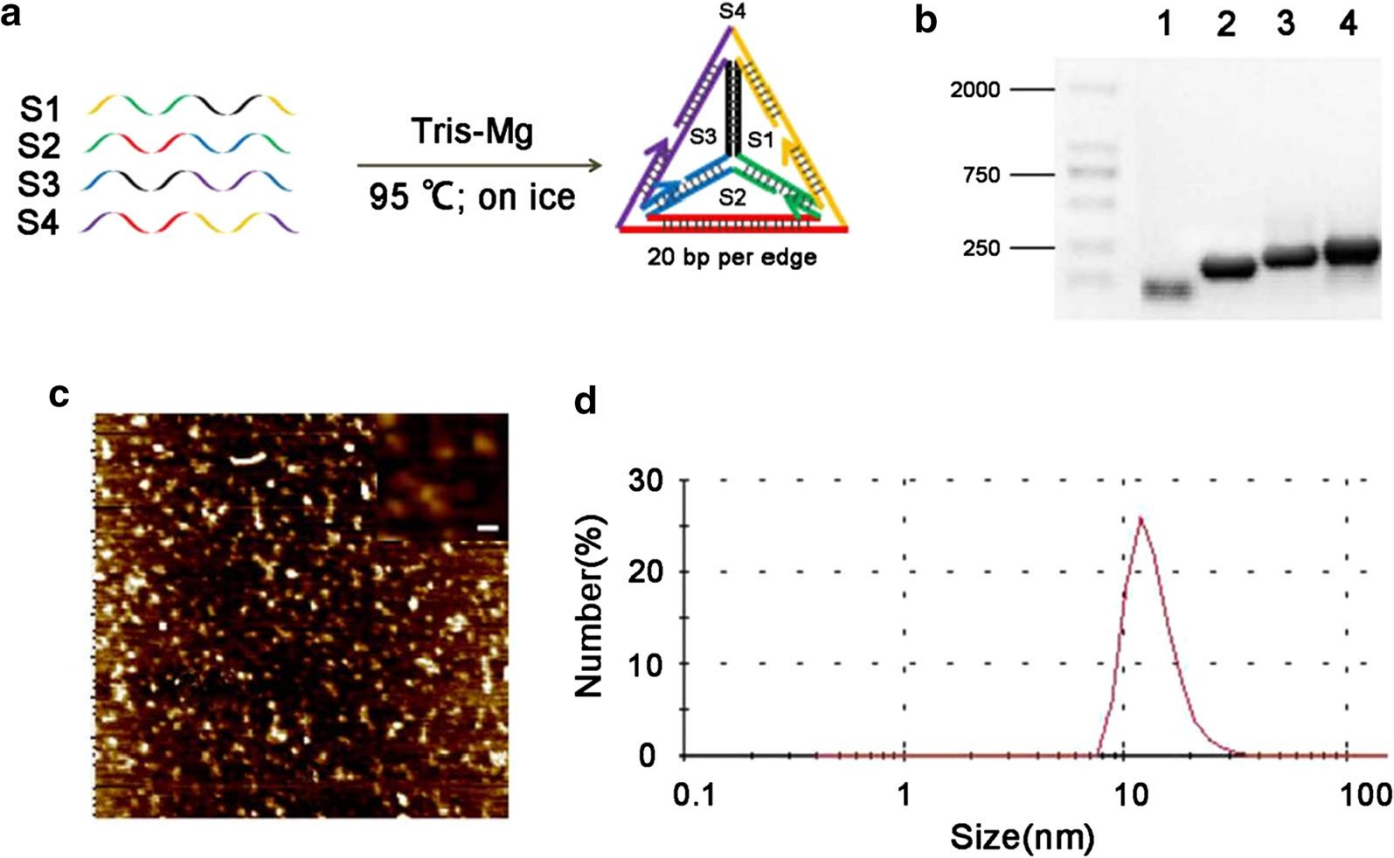
Characteristics of the Td. **a** A sketch of Td self-assembly. **b** Agarose gel of single-strand DNA, partial assembly, and Td. Lane 1: S1; Lane 2: S1 + S2; Lane 3: S1 + S2 + S3; Lane 4: S1 + S2 + S3 + S4. **c** AFM images of Td showing its triangle-like shape. The scale bar is 10 nm. **d** The hydrodynamic size of Td determined by DLS analysis

Td was prepared according to methods described previously [24] (Fig. 1a) and characterized by agarose gel, atomic force microscope (AFM) and dynamic light scattering (DLS). To verify the formation of Td, the four strands of the Td (S1, S2, S3, S4) were added one-by-one, and the gradually formed complex presented distinct bands with slower mobility as one more strand was added, indicating the successful stepwise assembly of the Td (Fig. 1b). AFM images showed that the Td exhibited an average diameter of approximately 10 nm, and a few aggregates were observed (Fig. 1c). DLS analysis revealed that the Td had a hydrodynamic size of ~ 12 nm with a polydispersity index (PDI) of 0.3 (Fig. 1d).

**Fig. 2:**
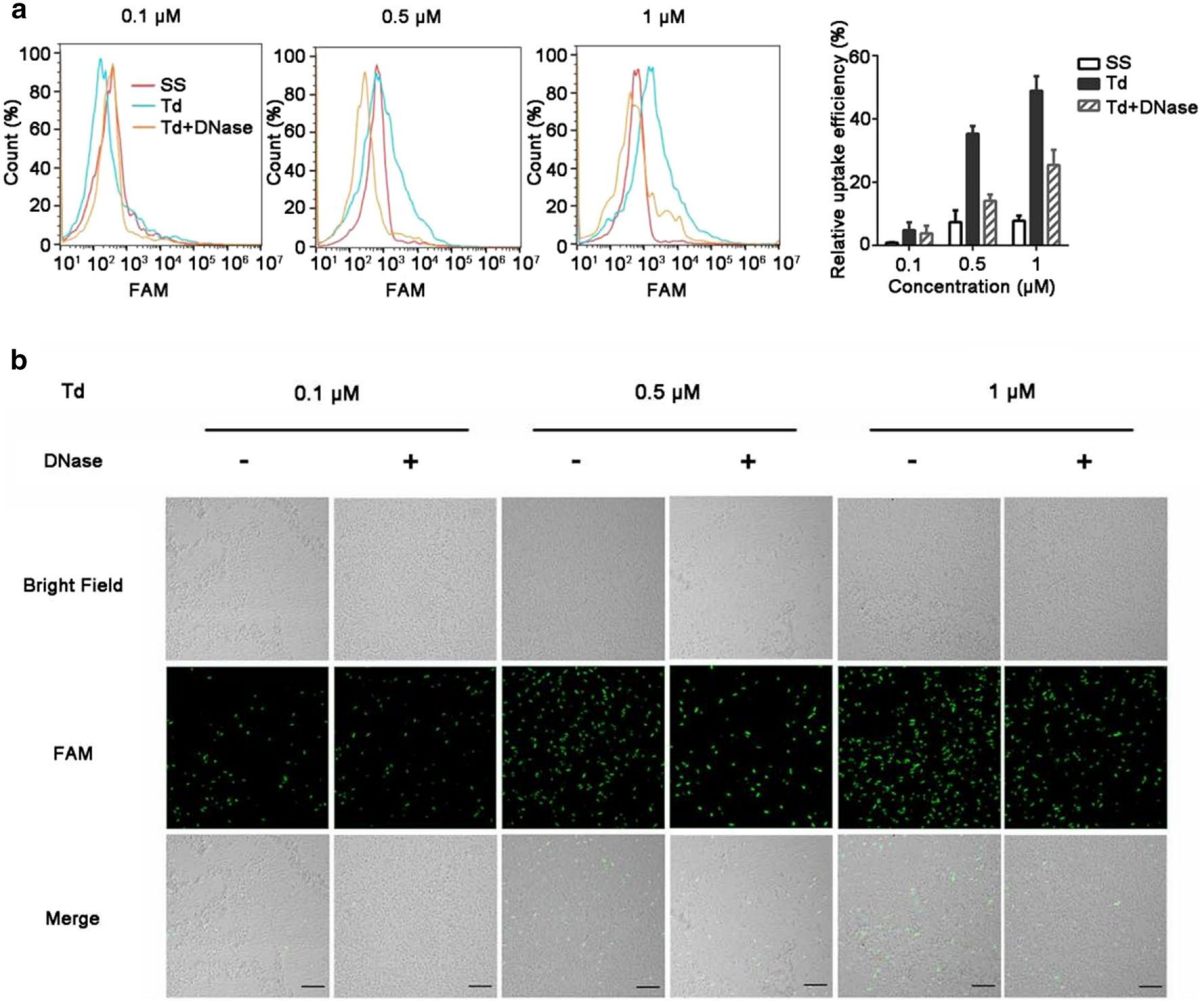
Td uptake by *E. coli*. **a** Flow cytometry and **b** confocal microscopy imaging to analyze the uptake efficiency of Td by *E. coli.* The bacterial cells were incubated with different concentrations of FAM-labeled Td (0.1, 0.5, or 1 μM) for 1.5 h and then were either treated or not treated with DNase before flow cytometry and confocal microscopy analyses. *SS* single-strand DNA, *Td* DNA tetrahedron. SS was used as a control. Scale bars represent 10 μm

Then, we studied the uptake features of Td in different bacteria. *E. coli* or *S. aureus* bacterial cells were incubated with FAM-labeled Td at various concentrations (0.1, 0.5, and 1 μM) for 1.5 h, and the number of FAM-positive bacteria was analyzed by flow cytometry. As a result, the positive ratios of *E. coli* incubated with FAM-labeled Td at concentrations of 0.1, 0.5, and 1 μM were 5%, 35%, and 49%, respectively (Fig. 2a), while the corresponding positive ratios of *S. aureus* were 5%, 24%, and 56% (Additional file 1: Figure S1a). To determine whether the observed fluorescence signals represented the uptake of the Td into bacteria or simple adherence to the bacterial membrane, the bacterial cells were treated with DNase before flow cytometry analysis [15]. And we demonstrated that DNase had no effect on FAM fluorescence intensity (Additional file 1: Figure S2). We found that after DNase treatment, the positive ratios of both tested strains decreased to approximately 20% when treated with 1 μM Td (Fig. 2a and Additional file 1: Figure S1a). However, the positive ratios of single-strand S1 labeled with FAM were lower than 5% in both tested bacteria whether treated with DNase or not (Fig. 2a and Additional file 1: Figure S1a). Furthermore, confocal laser scanning microscopy (CLSM) also confirmed that the fluorescence intensity of the tested bacteria was reduced significantly after treatment with DNase (Fig. 2b and Additional file 1: Fig. S1b). Similar results were also observed in other bacterial strains, including *S. flexneri*, *NDM1*-*K. pneumoniae*, *multiple*-*drug resistant P. aeruginosa* and *A. baumannii* (Additional file 1: Figure S3). All the data demonstrated that Td had a tendency to bond with the bacterial membrane and only a fraction of Td entered into bacterial cells successfully.

**Fig. 3:**
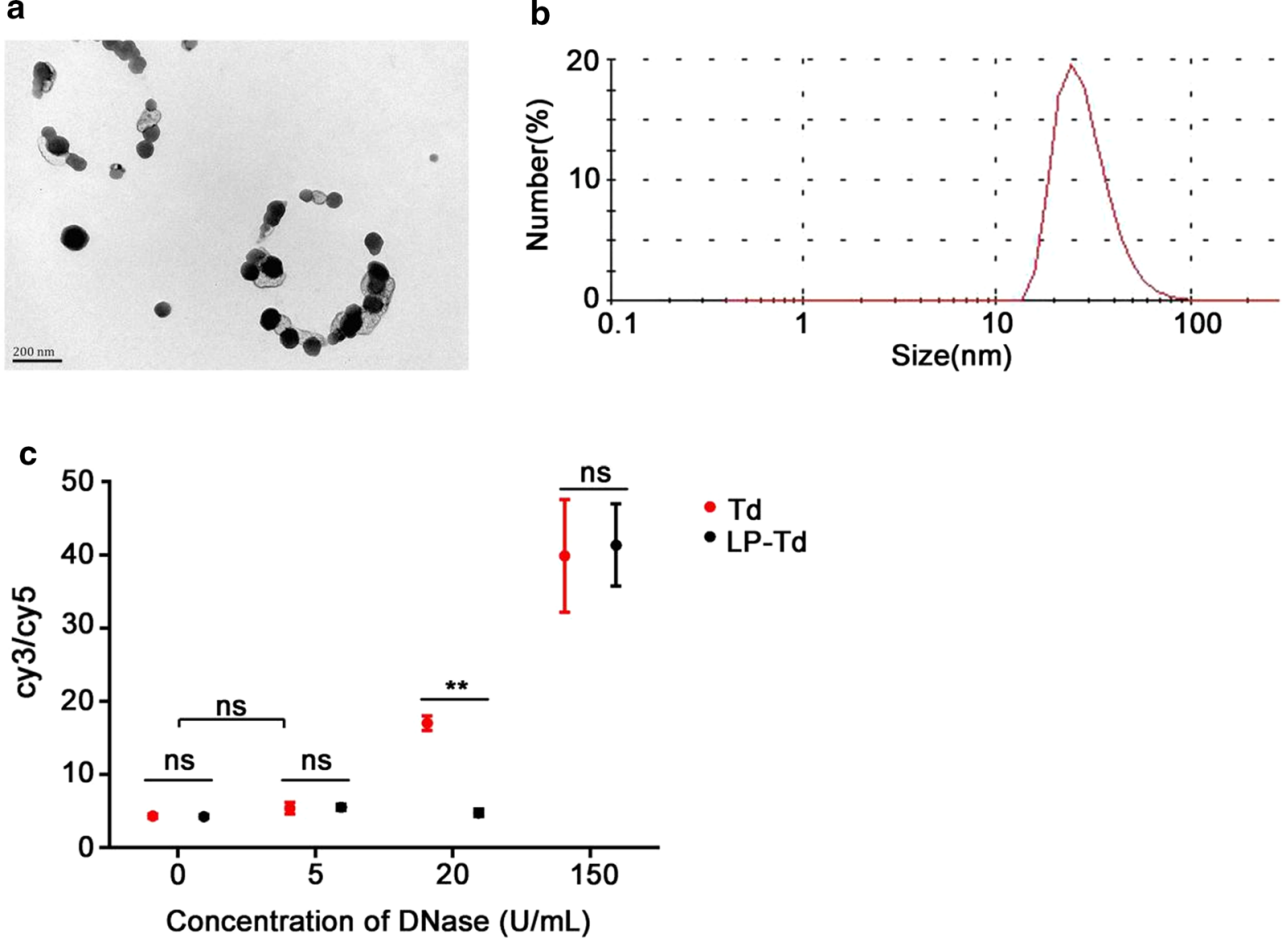
Characteristics and stability of the LP-Td. **a** TEM images of LP-Td. The scale bar is 200 nm. **b** The hydrodynamic size of LP-Td determined by DLS analysis. **c** Enzymatic stability of Td and LP-Td. 0.2 μM cy3/cy5 doubly labeled Td or LP-Td were incubated with DNase in different concentrations for 15 min. “ns”: no significance; “**”: *p* < 0.01

Next, Lipofectamine 2000 (LP2000) was used to improve the uptake efficiency of the Td by bacteria because of the following reasons: (1) We have demonstrated that lipofectamine 2000 (LP2000) could deliver oligonucleotides into bacteria effectively [25], and showed no toxicity when the concentration was lower than 10 μL/mL (Additional file 1: Figure S4). (2) Lipofectamine could interact with DNA by electrostatic force, and could be used as a modifier to increase the liposolubility of Td [26], which may help the DNA nanomaterials penetrate into bacterial cells more easily. Therefore, we explored the uptake efficiency of Td mixed with LP2000 (LP2000/Td: 0.0025, 0.0125, 0.025, and 0.125 μL/μg) for initial optimization. The Td mixed with LP2000 (LP-Td) showed the same size as the Td when the LP2000/Td ratio was 0.0025 (Additional file 1: Figure S5a), while larger nanoparticles were formed when the ratios were greater than 0.0025. And the size increased as the ratio increased (Additional file 1: Figure S5b, c). However, when the ratio of LP2000/Td was 0.125 μL/μg, the formed nanoparticles exhibited homogeneous sizes of approximately 30 nm (Additional file 1: Figure S5d). Then we used this ratio (0.125 μL/μg) to assemble LP-Td, and characterized it by TEM and DLS (Fig. 3a, b). To further investigate whether LP2000 at this concentration could protect Td from enzymatic hydrolysis, structural integrity of Td and LP-Td was monitored by FRET [15]. Strand S2 and S3 were labeled with cy3 and cy5 respectively, which were close enough for energy transfer from the donor cy3 to the acceptor cy5 when Td was intact. Once Td was degraded, a donor (cy3) was separated from an acceptor (cy5), then the fluorescence intensity of cy5 decreased while that of cy3 increased. Figure 3c showed that although Td and LP-Td were both degraded in 150 U/mL DNase, LP2000 protected Td from hydrolysis when DNase was 20 U/mL. This data indicated that LP-Td had higher enzyme stability than Td to some extent.

**Fig. 4:**
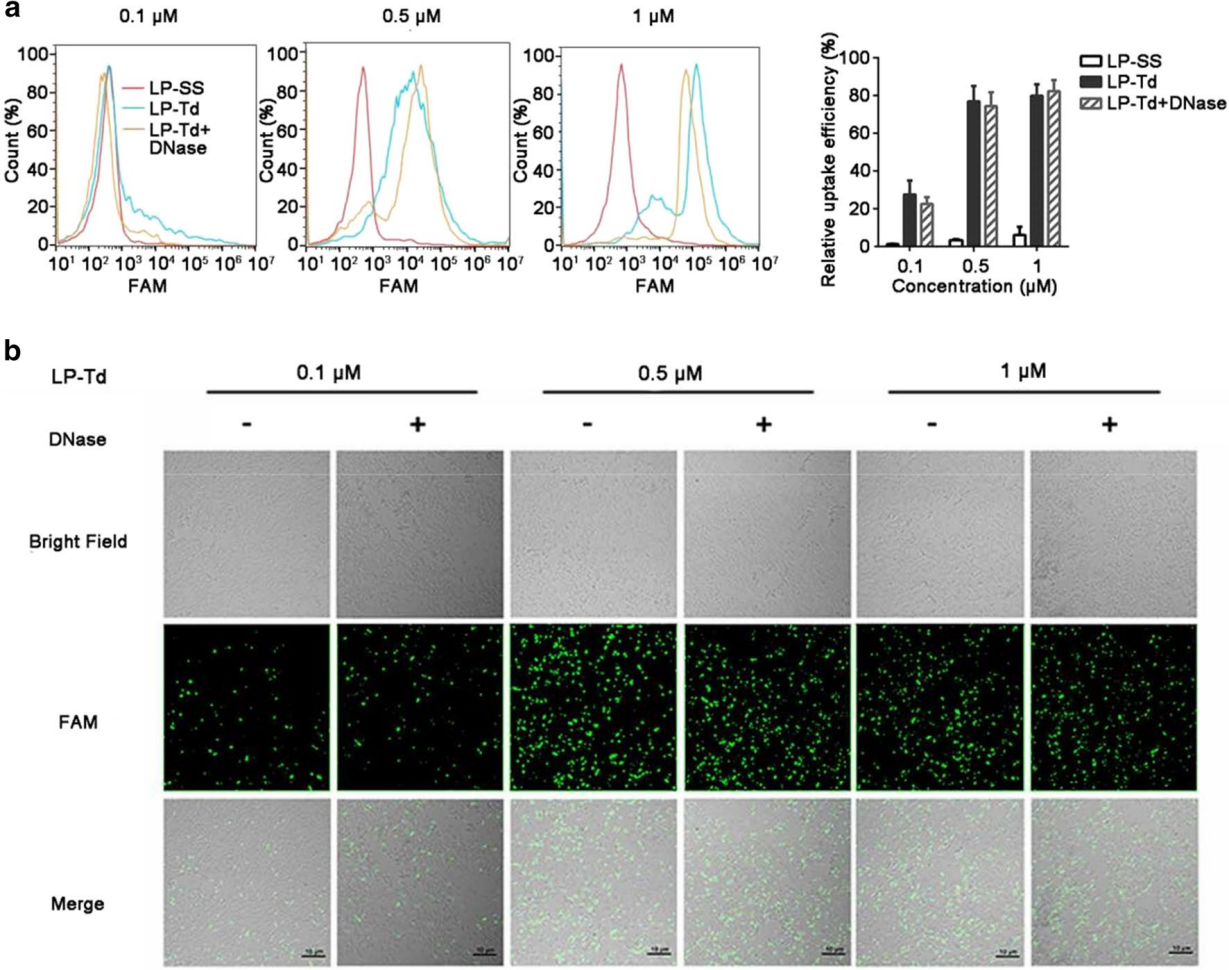
Td uptake by *E. coli* with the help of LP2000. **a** Flow cytometry and **b** confocal microscopy imaging to analyze the uptake efficiency of Td mixed with LP2000 (LP-Td) by *E. coli.* The bacterial cells were incubated with different concentrations of FAM-labeled LP-Td (0.1, 0.5, or 1 μM) for 1.5 h and then either treated or not treated with DNase before flow cytometry and confocal microscopy analyses. The LP2000/Td ratio was 0.125 μL/μg. SS: single-strand DNA; Td: DNA tetrahedron; LP: LP2000. SS was used as a control. Scale bars represent 10 μm

According to the results of flow cytometry and CLSM, the positive ratio in *E. coli* improved gradually as an increased amount of LP2000 was added, reaching 83% when the concentration of Td was 0.5 μM and the LP2000/Td ratio was 0.125 μL/μg (Additional file 1: Figure S6a, b). However, increasing the LP2000/Td ratio only slightly improved the positive ratio of Td in *S. aureus*, reaching a peak value of only 40%, even at the highest LP2000/Td ratio (Additional file 1: Figure S6c, d). This result may be attributed to the existence of more peptidoglycan in Gram-positive bacteria, which hindered the interaction between LP-Td and the lipid membrane. Strikingly, bacteria treated with single-strand DNA exhibited very low positive ratios (< 10%) regardless of how much LP2000 was added in those ratios. However, because the difference of molecular weights between Td and single-strand DNA led to distinct absolute amounts of LP2000, we added the same amounts of LP2000 (0.125 μL/μg to 1 μM Td) to 1 μM single-strand DNA and then incubated with *E. coli* and *S. aureus*, to eliminate this interference and further clarify the superiority of Td to enter bacteria. The positive ratios were 20% and 18% in *E. coli* and *S. aureus*, respectively (Additional file 1: Figure S7a). Importantly, when LP2000 with a 0.125 μL/μg ratio to Td was added, DNase treatment had no influence on the ratio of FAM-positive bacteria, as demonstrated by the results of flow cytometry (Fig. 4a and Additional file 1: Figure S8a) and CLSM image analysis (Fig. 4b and Additional file 1: Figure S8b). Furthermore, Triton X-100 was used to disrupt LP-Td nanoparticles which may adhere to bacterial membrane, and the positive ratios did not decrease (Additional file 1: Figure S7b). All results indicated that most of the Td crossed the membrane and entered bacteria with the help of LP2000. To fit a dose response curve for uptake efficiency, *E. coli* or *S. aureus* were incubated with 0.1, 0.2, 0.3, 0.4, 0.5, 0.6, 0.7, 0.8, 0.9 or 1 μM FAM-labeled LP-Td for 1.5 h, and then the positive ratios were tested by Flow cytometry. Graphpad prism was used to fit a curve, which was shown in Additional file 1: Figure S9, and the dose-responsive equations for uptake efficiency were obtained. *E. coli*: Y = 22 + (79.93 − 22)/(1 + 10^((− 0.4657-X)*9.243)); *S. aureus*: Y = 28.54 + (91.12 − 28.54)/(1 + 10^((− 0.2352-X)*2.919)). And the EC_50_ for *E. coli* and *S. aureus* were 0.34 and 0.58 μM respectively. Y means relative uptake efficiency (%), and X means the concentration of LP-Td.

Next, the influences of incubation temperature and time on the uptake efficiency of LP-Td were also investigated. As a result, the uptake efficiency did not decrease when *E. coli* and *S. aureus* were treated with LP-Td at 4 °C compared to 37 °C, indicating that the uptake process in bacteria was energy-independent (Additional file 1: Figure S10a). After incubation with 0.5 μM LP-Td for 10, 30, 60, 180 min, we found that the uptake efficiencies in *E. coli* and *S. aureus* reached the maximum value at the first time point (10 min) and remained at relatively constant values at longer incubation times, indicating that the process of LP-Td internalization in bacteria was fairly quick (less than 10 min) (Figure S10b). As a transfection reagent, LP2000 is commonly used in eukaryotic cells with an LP2000/DNA ratio of 2–3 μL/μg. In the present study, much less LP2000 (with an LP2000/Td ratio of 0.125) could significantly facilitate bacterial uptake of Td; by contrast, this amount of LP2000 had no effect on the uptake of single-strand DNA. Based on these results, we speculate that in this uptake process, the unique structure of the Td increases its binding with bacterial surface, while the hydrophobicity of LP2000 increases the transmembrane ability of the Td. Therefore, LP-Td could be successfully internalized by bacteria. Furthermore, the quantity of LP2000 used here (1 μM Td; 0.125 μL/μg LP2000/Td ratio, corresponding to 10 μL/mL LP2000) exerted no toxicity on *E. coli* and *S. aureus*. In fact, *E. coli* could endure higher concentrations of LP2000 (Additional file 1: Figure S4).

**Fig. 5:**
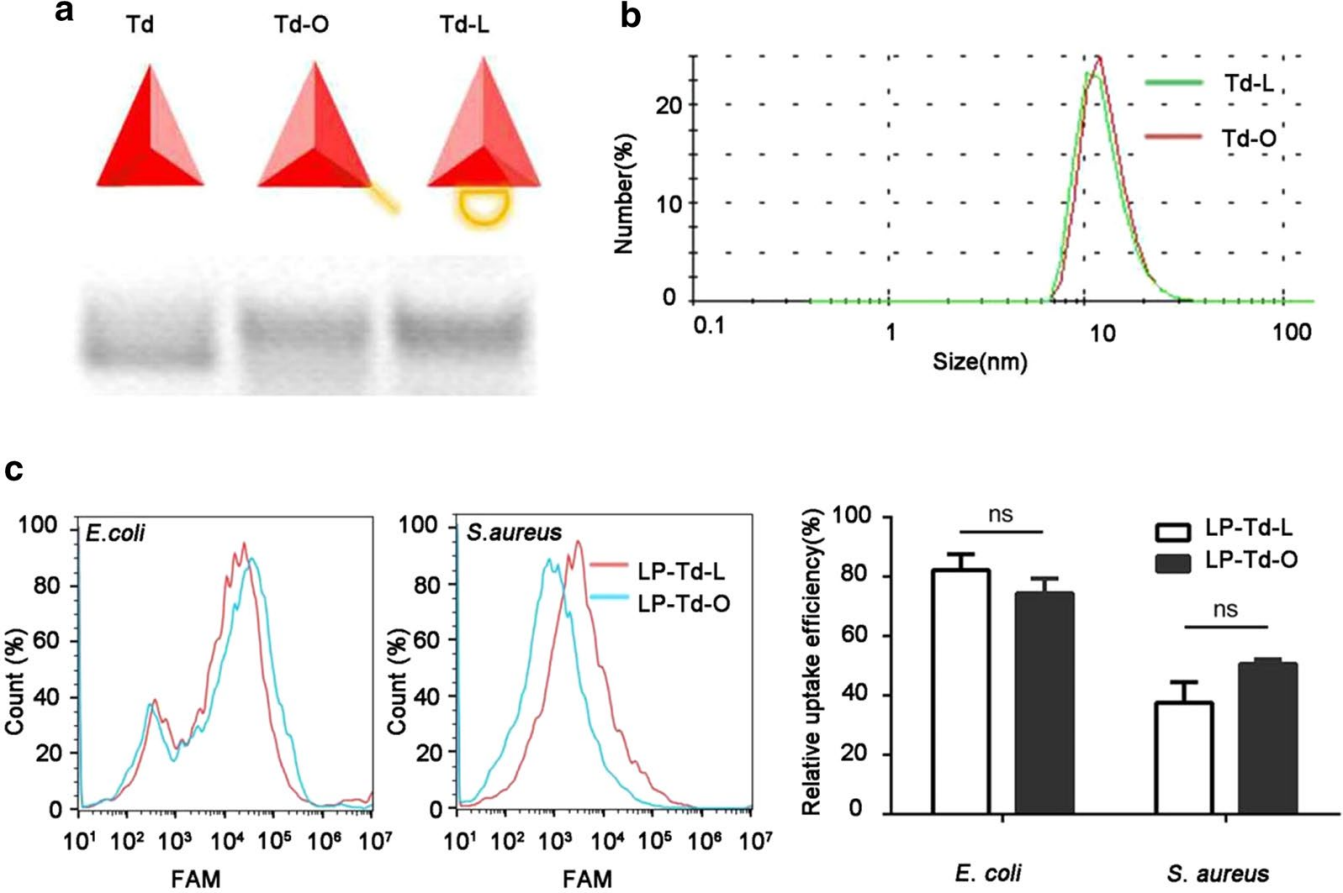
Characteristics and uptake efficiency of Td carrying ASOs. **a** A sketch of Td-O and Td-L and the formation of Td-O and Td-L verified by agarose gel. **b** The hydrodynamic size of Td-O and Td-L determined by DLS analysis. **c** Flow cytometry analysis of the uptake efficiency of LP-Td-O and LP-Td-L by *E. coli* and *S. aureus*. The bacterial cells were incubated with 0.5 μM FAM-labeled LP-Td-L or LP-TD-O for 1.5 h and then assessed by flow cytometry. LP: LP2000

To investigate whether the LP-Td complex could deliver ASOs into bacteria and exert gene inhibitory effects, two different strategies were designed to link functional ASOs to the Td: 1) Td-L, in which a single strand was protruding from the side of the Td as a loop (Td-L) (with 3 additional nucleotides at the end of both linking sites to expose antisense sequences), and 2) Td-O, in which the single strand was overhanging at one vertex of the Td (with 7 additional nucleotides added to the end of linking site to expose antisense sequences) (Fig. 5a). To improve stability, ASOs were modified by phosphorothioate. The agarose gel results indicated the successful formation of both structures, which exhibited distinct bands with a slightly slower mobility than Td (Fig. 5b). DLS demonstrated that the sizes of Td-L and Td-O were similar to that of Td (Fig. 5c) and increased to ~ 30 nm when LP2000 was added (Figure S11).

**Fig. 6:**
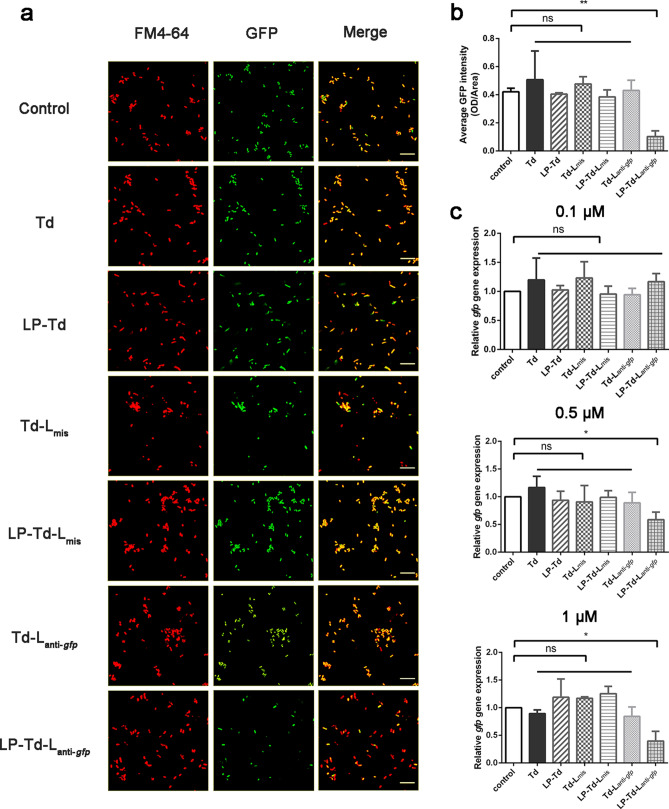
Gene-inhibiting efficiency of LP-Td-L targeting *gfp* in *E. coli* expressing GFP. **a** Confocal microscopy imaging to investigate GFP fluorescence intensity after treating bacteria with 1 μM Td-L_anti-*gfp*_ with or without LP2000 for 6 h. Red fluorescence was from FM4-64. **b** Semiquantitative analysis of GFP fluorescence intensity. **c** Quantitative real-time PCR analysis of *gfp* mRNA levels after exposure to different concentrations of Td-L_anti-*gfp*_ or LP-Td-L_anti-*gfp*_ (0.1, 0.5, or 1 μM) for 3 h. Td-L_mis_ was used as a negative control to confirm specific gene inhibiting of ASOs. Scale bars represent 10 μm. “*”: *p* < 0.05; “**”: *p* < 0.01 versus control group

**Fig. 7:**
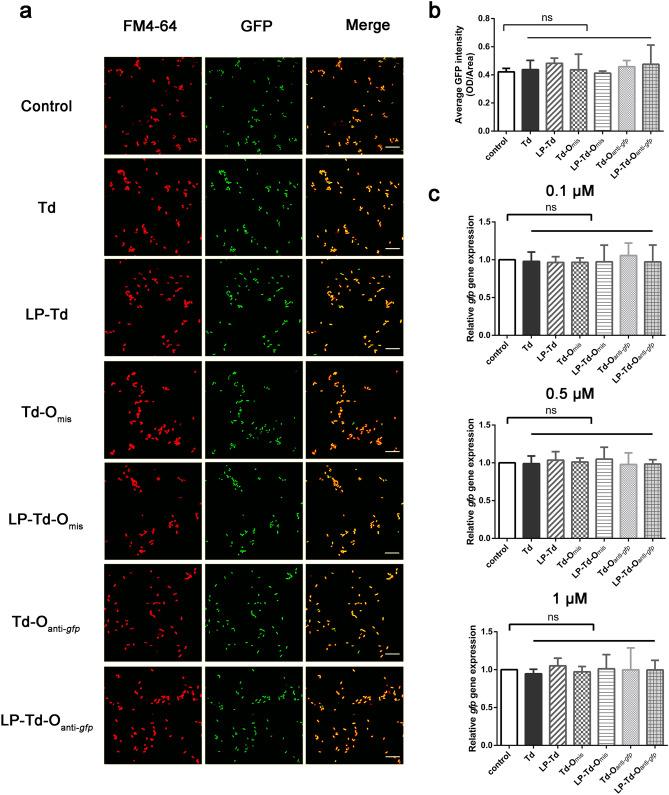
Gene-inhibiting efficiency of LP-Td-O targeting *gfp* in *E. coli* expressing GFP. **a** Confocal microscopy imaging to investigate GFP fluorescence intensity after treating bacteria with 1 μM Td-O_anti-*gfp*_ with or without LP2000 for 6 h. Red fluorescence was from FM4-64. **b** Semiquantitative analysis of GFP fluorescence intensity. **c** Quantitative real-time PCR analysis of *gfp* mRNA levels after exposure to different concentrations of Td-O_anti-*gfp*_ or LP-Td-O_anti-*gfp*_ (0.1, 0.5, or 1 μM) for 3 h. Td-O_mis_ was used as a negative control to confirm specific gene inhibiting of ASOs. Scale bars represent 10 μm. “*”: *p* < 0.05; “**”: *p* < 0.01 versus control group

Next, the gene-inhibiting effects of Td-L and Td-O carrying anti-*gfp* ASOs were studied in *E. coli* expressing GFP (GFP-*E. coli*). Mismatched ASOs linked to Td (Td-L_mis_ and Td-O_mis_) were used as controls to confirm the specific gene-inhibiting effect. A confocal study showed that Td-L_anti-*gfp*_, Td and Td-L_mis_ at a concentration of 1 μM all had no influence on the fluorescent intensity of GFP-*E. coli* (Fig. 6a, b). In contrast, when treated with LP-Td-L_anti-*gfp*_ (1 μM), a 75% reduction in GFP fluorescence intensity was observed in GFP-*E. coli*, while LP-Td and LP-Td-L_mis_ also had no effect (Fig. 6a, b). However, unlike Td-L_anti-*gfp*_, Td-O_anti-*gfp*_ did not affect GFP fluorescence regardless of whether LP2000 was used (Fig. 7a, b), implying the importance of the specific structure of Td-L for the gene inhibitory effect.

Then, we tested whether the observed decrease in fluorescence intensity in GFP-*E. coli* resulted from targeted gene inhibition. After incubating for 3 h, bacteria were collected, and total RNA was extracted to detect the mRNA level of *gfp*. The results in Fig. 6c show that when treated with LP-Td-L_anti-*gfp*_ at concentrations of 0.5 and 1 μM, *gfp* expression decreased by 41.5% and 60.5%, respectively, while treatment with 0.1 μM LP-Td-L_anti-*gfp*_ did not lead to an observable decrease in *gfp* expression. Moreover, no significant decreases in *gfp* mRNA level were observed in the other groups at all concentrations. In addition, Fig. 7c indicates that Td-O_anti-*gfp*_ and LP-Td-O_anti-*gfp*_ did not inhibit the *gfp* expression level, consistent with the confocal results. However, flow cytometry analysis demonstrated that the bacterial uptake efficiencies of Td-L and Td-O were comparable (Fig. 5d), indicating that the loop structure in Td-L facilitated its gene inhibition activity. The loop design may help ASOs combine with RNA or increase the sensitivity of the ASOs/RNA complex to RNase. Further studies to uncover the underlying mechanisms of the antisense effect of Td-L can provide potential strategies to design more effective linkage modes between Td and ASOs.

**Fig. 8:**
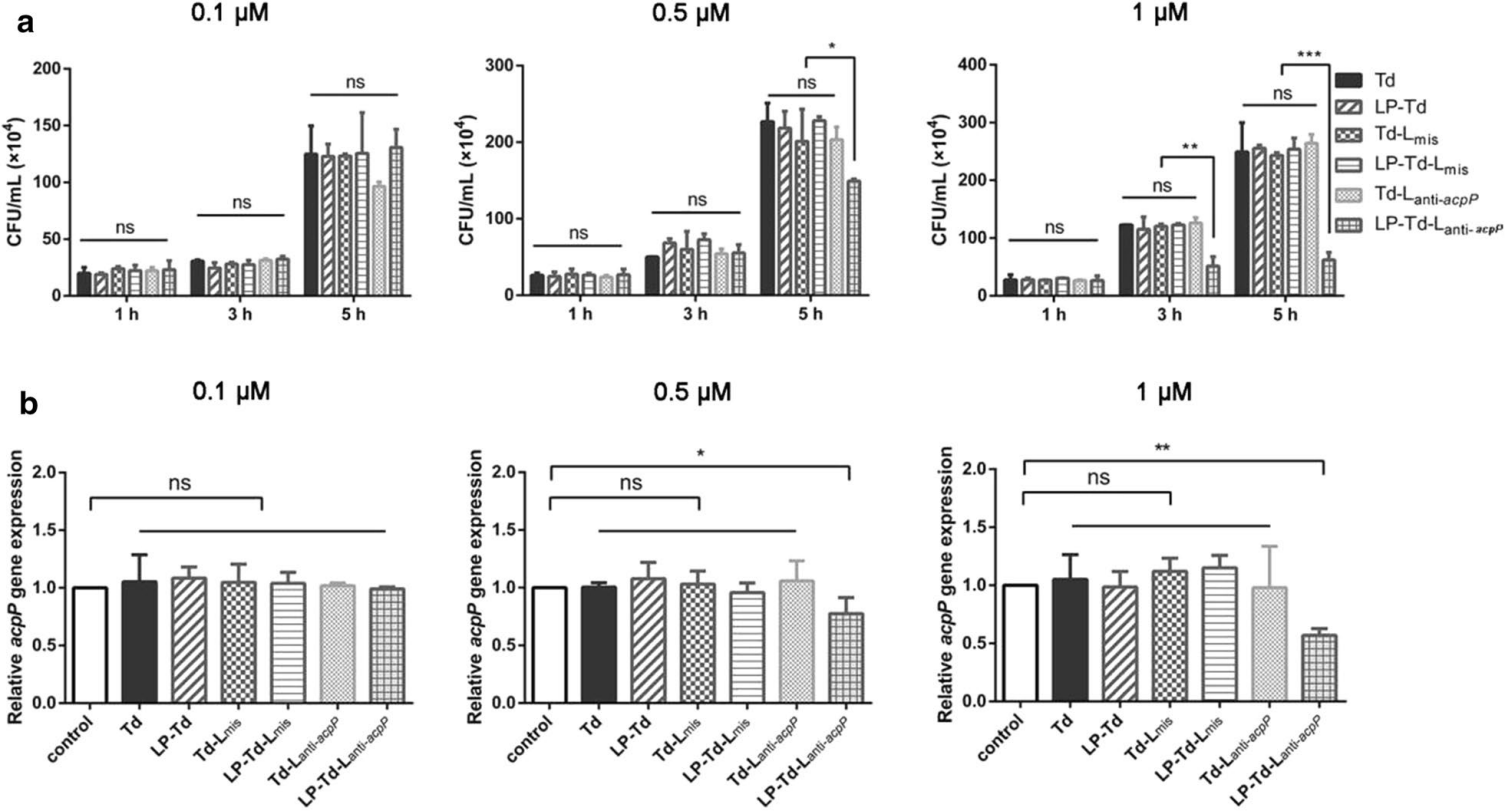
Bacterial growth inhibitory effect of LP-Td-L by inhibiting the *acpP* gene in *E. coli*. **a** Colony counting of *E. coli* after treatment with different concentrations of Td-L_anti-*acpP*_ (0.1, 0.5, or 1 μM) with or without LP2000 for different times (1, 3 or 5 h). **b** Quantitative real-time PCR analysis of *acpP* mRNA levels after exposure to different concentrations of Td-L_anti-*acpP*_ or LP-Td-L_anti-*acpP*_ (0.1, 0.5, or 1 μM) for 3 h. Td-L_mis_ was used as a negative control to confirm specific gene inhibiting of ASOs. “*”: *p* < 0.05; “**”: *p* < 0.01; “***”: *p* < 0.001 versus control group

Finally, we investigated the antibacterial activity of Td-L carrying anti-*acpP* ASOs (Td-L_anti-*acpP*_). Here, *acpP* is a gene encoding the acyl carrier protein Acp, which is critical for fatty acid biosynthesis in *E. coli*. As shown in Fig. 8a, 0.1 μM LP-Td-L_anti-*acpP*_ did not influence the growth of *E. coli*. When the concentration was increased to 0.5 μM, LP-Td-L_anti-*acpP*_ significantly inhibited the growth of *E. coli* at 5 h after treatment, as highlighted by the reduced colony-forming units (CFU) in the LP-Td-L_anti-*acpP*_ group compared to the other groups. Furthermore, 1 μM LP-Td-L_anti-*acpP*_ exhibited inhibitory effects on bacterial growth at 3 h and stronger effects at 5 h. Then, we further analyzed the mRNA level of *acpP* to examine whether the LP-Td-L_anti-*acpP*_-induced bacterial inhibition was mediated by targeting *acpP.* As a result, the mRNA level of *acpP* was found to significantly decrease in the LP-Td-L_anti-*acpP*_ group compared to the other groups. The mRNA expression level of *acpP* decreased by 23% and 43% after treatment with 0.5 μM and 1 μM LP-Td-L_anti-*acpP*_, respectively (Fig. 8b). Collectively, these results demonstrated that LP-Td-L_anti-*acpP*_ exhibited antibacterial activity via its gene inhibitory effects targeting *acpP* and that LP2000 played a fairly essential role in the process.

## Conclusions

In this study, we investigated the uptake efficiency of DNA tetrahedron (Td) in different bacterial strains. Interestingly, the Td adhered to the surface of the bacterial membrane efficiently, but a few could penetrate the membrane, probably due to its hydrophilicity. However, a very low ratio of LP2000 to Td facilitated the penetration of the Td into bacteria within a few minutes in an energy-independent manner. Compared with single strand DNA, Td showed superiority of crossing bacterial membrane with the help of LP2000, the reason of which may be its characteristic structure. In addition, based on the optimized LP-Td system, the modes of ASOs linked with the Td had a significant impact on gene knockdown efficiency, with the looped structure of ASOs linked to one side of Td exhibiting excellent gene-inhibiting activity. Importantly, LP-Td-L_anti-*acpP*_ showed gene inhibitory effects targeting the *acpP* gene and eventually inhibited bacterial growth. Therefore, this study provides novel insights into the uptake features of Td in bacteria and highlights the importance of linkage strategy between ASOs and Td. In further research, multivalent ASO-containing particles could be designed based on the superior structure of Td and the selectivity of ASOs linking modes, which will open novel opportunities for developing effective antisense delivery systems.

## Material and method

### Materials

All oligonucleotides were obtained from Sangon Biotech (China). Magnesium chloride hexahydrate was purchased from Tian Li (China). Trizma base was obtained from Sigma-Aldrich (USA). Agarose was purchased from Lonza (USA). DNase I was obtained from Solarbio (China). Lipofectamine 2000 (LP2000) was purchased from Invitrogen (USA). FM4-64 was purchased from Molecular Probes (USA). Phosphate-buffered saline (PBS) and Mueller–Hinton broth (MHB) were purchased from Boster (China) and Land Bridge (China), respectively. The PrimeScript RT Reagent Kit with DNA Eraser and Premix Taq RT-PCR System were from Takara Bio Inc (Japan). Thse water used in all experiments was prepared via a Millipore Milli-Q purification system with a resistivity greater than 18 MΩ cm^−1^.

### Methods

#### Self-assembly of Td

All single strands(S1, S2, S3, S4) in equimolar quantities were mixed in TM buffer (12.5 mM Tris, 5 mM MgCl_2_, pH = 7.8–8.0), heated to 95 °C for 5 min with an MJ MiniTM 48-well personal thermal cycler, and then cooled rapidly on ice for at least 1 h.

#### Preparation of LP-Td

Td and LP2000 were diluted in PBS respectively, and mixed. Then the mixture was incubated for 15 min at room temperature to form the LP-Td.

#### Agarose gel electrophoresis

A 1% (w/v) agarose gel was prepared with EtBr. A total of 5 μL prepared Td mixed with loading buffer was loaded and run at 95 V for 35 min. To identify the Td with ASOs, a 2% (w/v) agarose gel was used.

#### AFM imaging

All samples were diluted to 2.5 nM in TM buffer (12.5 mM Tris, 5 mM MgCl_2_). Then, 10 μL of Td was dropped onto freshly cleaved mica and incubated for 5 min to allow strong absorption onto the surface. Then, the mica was rinsed using filtered deionized water and gently dried with compressed nitrogen. Next, the samples were scanned in tapping mode on an Agilent 5500 SPM.

#### Dynamic light scattering

A 2 μM solution of Td was prepared based on the above-mentioned protocols and then diluted to 0.2 μM with ultrapure water. After passing through a 0.22 μm filter, samples were analyzed by a Malvern Zetasizer Nano ZS to measure the hydrodynamic size and size distribution.

#### TEM

LP-Td was dropped on a grid and incubated for 4 min, then remaining solution was absorbed by filter paper. The TEM images were obtained by JEM-2100Plus with 80 kV.

#### Enzymatic stability experiment

Td was self-assembled with S1, cy5-S2, cy3-S3 and S4. Then the fluorescence of 0.2 μM Td or LP-Td was measured by F-2500 FL Spectrophotometer after incubation with 0, 5, 20, 150 U/mL DNase for 15 min. The fluorescence intensity of 560 nm (cy3) and 665 nm (cy5) was recorded.

#### Transfection of bacteria

FAM-labeled S1 was used for the preparation of fluorescently labeled Td. Bacteria (1 × 10^6^ CFU/mL) were incubated with 0.1, 0.5, or 1 μM FAM-Td in PBS and then treated with 500 U/mL DNase for 15 min. FAM-S1 was used as a control. To optimize the transfection conditions, LP2000 was mixed with Td at ratios of 0.0025, 0.0125, 0.025, and 0.125 μL/μg before incubating with bacteria, and the optimum ratio (0.125 μL/μg) was used in subsequent experiments.

#### Flow cytometry

The uptake efficiency of Td in bacteria was estimated by flow cytometry. The transfected samples were centrifuged at 6000 g/min for 5 min and washed twice with PBS. After resuspending in PBS, the samples were detected with a blue laser (488 nm excitation) and a 530/30 filter in the BL1 detector by a NovoCyte™ flow cytometer.

#### Confocal microscopy

Bacteria were stained with 20 g/mL FM4-64 on ice for 1 min, then centrifuged and resuspended in PBS again. Then, 3.5 μL of sample was dropped onto a slide and dried in air. Next, 5 μL of antifade mounting medium was added and covered with a coverslip. All images were obtained using an Olympus FluoView™ FV1000 microscope. The set wavelengths were 543 nm excitation for FM4-64 and 488 nm excitation for FAM and GFP.

#### Cell growth assay

*Escherichia coli* bacteria were cultured in LB medium until the early log stage, then diluted to 1 × 10^5^ CFU/mL and treated with Td-L at 0.1, 0.5, or 1 μM for 1, 3, or 5 h. At every time point, a 10 μL bacterial suspension was diluted and plated. After incubating at 37 °C overnight, colony counts in every sample were expressed as CFU/ml.

#### RT-PCR

After incubation with Td, the bacteria were harvested. The total RNA of the bacteria was extracted and then reverse-transcribed using the PrimeScript RT Reagent Kit with DNA Eraser. Amplification was performed according to the following steps: denaturation at 95 °C for 5 min and 40 cycles of 95 °C for 10 s and 58 °C for 30 s. gyrA was used as an internal control.

#### Statistical analysis

The results are shown as the mean ± SD from at least three independent experiments. Statistical analyses were suitably implemented in Prism 6 (GraphPad, La Jolla, CA) with one-way ANOVA. A *p* value < 0.05 indicated a significant difference between group means.

## Supplementary information

**Additional file 1.** Additional figures.

## Data Availability

All data generated or analyzed during this study are included in this published article.
