# Supplementary material for: Loop-armed DNA tetrahedron nanoparticles for delivering antisense oligos into bacteria
Source: J Nanobiotechnology. 2020 Aug 4;18:109. doi: 10.1186/s12951-020-00667-6 (PMC7401225; doi:10.1186/s12951-020-00667-6)
Supplement: Supplementary file 1 — Additional file 1. Additional figures. [file 12951_2020_667_MOESM1_ESM.docx]

Additional Information

Loop-armed DNA Tetrahedron Nanoparticles for Delivering Antisense Oligos into Bacteria

Yue Hu^†, 1^, Zhou Chen^†, 1^, Xinggang Mao^‡, 1^, Mingkai Li^†^, Zheng Hou^†^, Jingru Meng^†^, Xiaoxing Luo^†,*^, Xiaoyan Xue^†,*^

**DNA strands for self-assembly**

S1 AGGCAGTTGAGACGAACATTCCTAAGTCTGAAATTTATCACCCGCCATAGTAGACGTATCACC

S2 CTTGCTACACGATTCAGACTTAGGAATGTTCGACATGCGAGGGTCCAATACCGACGATTACAG

S3 GGTGATAAAACGTGTAGCAAGCTGTAATCGACGGGAAGAGCATGCCCATCCACTACTATGGCG

S4 CCTCGCATGACTCAACTGCCTGGTGATACGAGGATGGGCATGCTCTTCCCGACGGTATTGGAC

Loop-S1

TTTATCACCCGCCATAGTAGACGTATCACCAGGCAGTTGAGA

Overhang-S1

CGAACATTCCTAAGTCTGAAATTTATCACCCGCCATAGTAGA

Loop_anti-_*_gfp_*

CGAACATTCCTTTACAGCTCCTCGCCCTTCGTTTTAAGTCTGAAA

Overhang_anti-_*_gfp_*

CGTATCACCAGGCAGTTGAGATTTTTTTACAGCTCCTCGCCCTTCG

Loop_anti-_*_acpP_*

CGAACATTCCTTTCTTCGATAGTGTTTTAAGTCTGAAA

Overhang_anti-_*_acpP_*

CGTATCACCAGGCAGTTGAGATTTTTTTCTTCGATAGTG

Loop_mis_

CGAACATTCCTTTATCGGCCATCGCCATCCGTTTTAAGTCTGAAA

Overhang_mis_

CGTATCACCAGGCAGTTGAGATTTTTTTATCGGCCATCGCCATCCG

**Plasmid profile of pEGFP-LAC**

pEGFP-LAC was purchased from Miaolingbio (China).

**
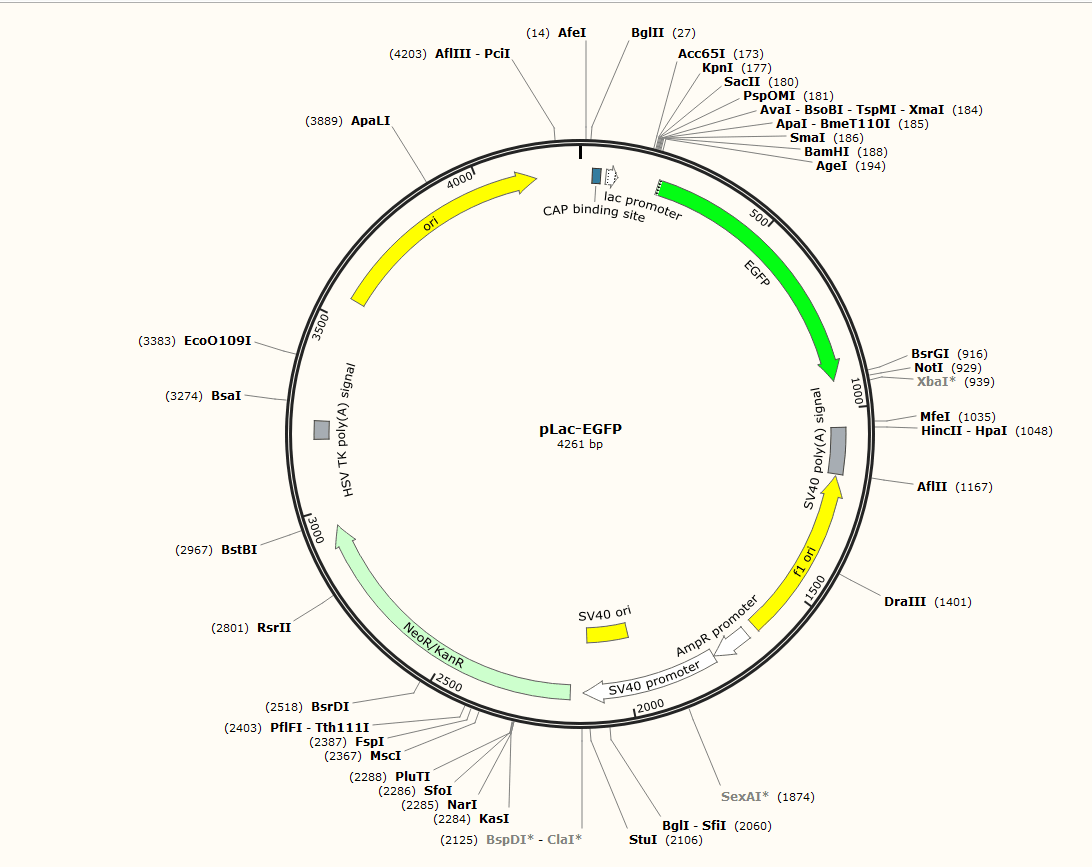
**

**
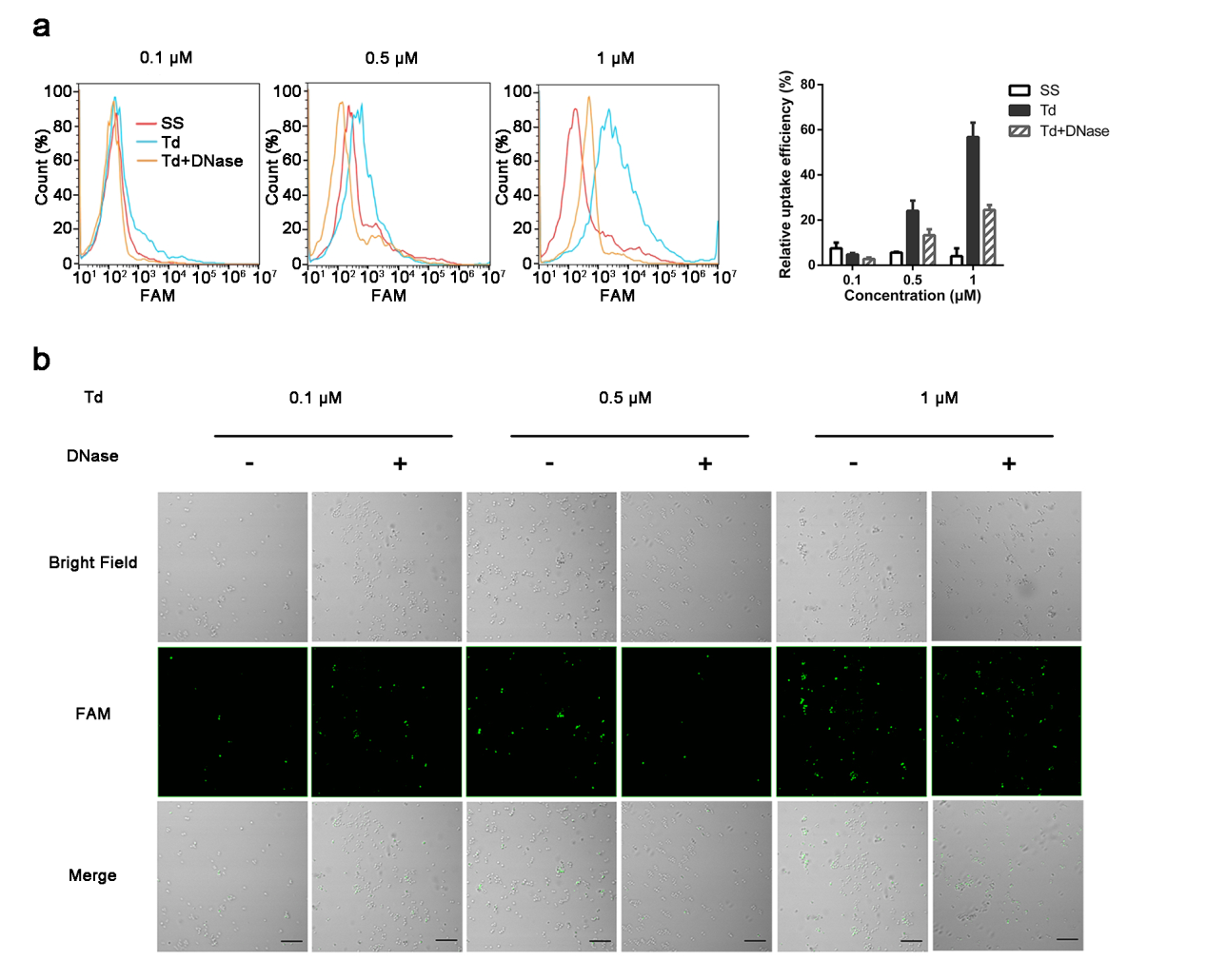
**

**Figure S1. Td uptake by *S.aureus*.** a) Flow cytometry and b) confocal microscopy imaging to analyze the uptake efficiency of Td by *S.aureus.* The bacterial cells were incubated with different concentrations of FAM-labeled Td (0.1, 0.5, or 1 μM) for 1.5 h and then were either treated or not treated with DNase before flow cytometry and confocal microscopy analyses. SS: single-strand DNA; Td: DNA tetrahedron. SS was used as a control. Scale bars represent 10 μm.


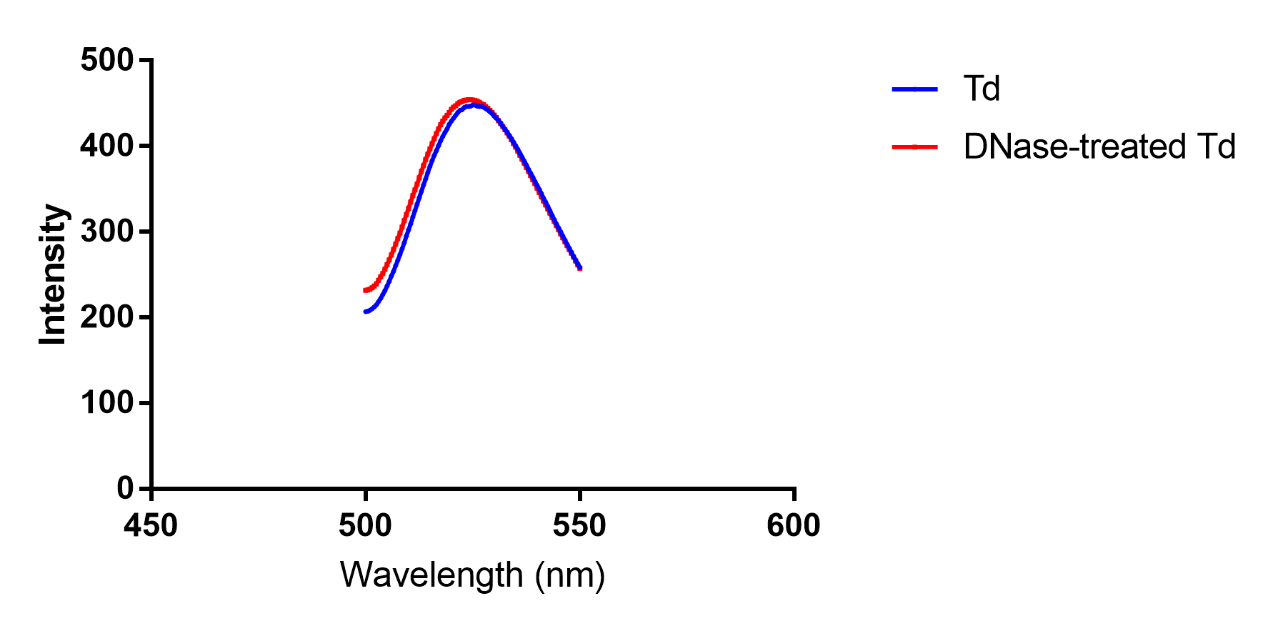


**Figure S2. Effect of DNase on FAM fluorescence.** 0.5 μM FAM-labeled DNA was incubated with or without DNase for 60 min, and then measured the FAM fluorescence intensity respectively.

**
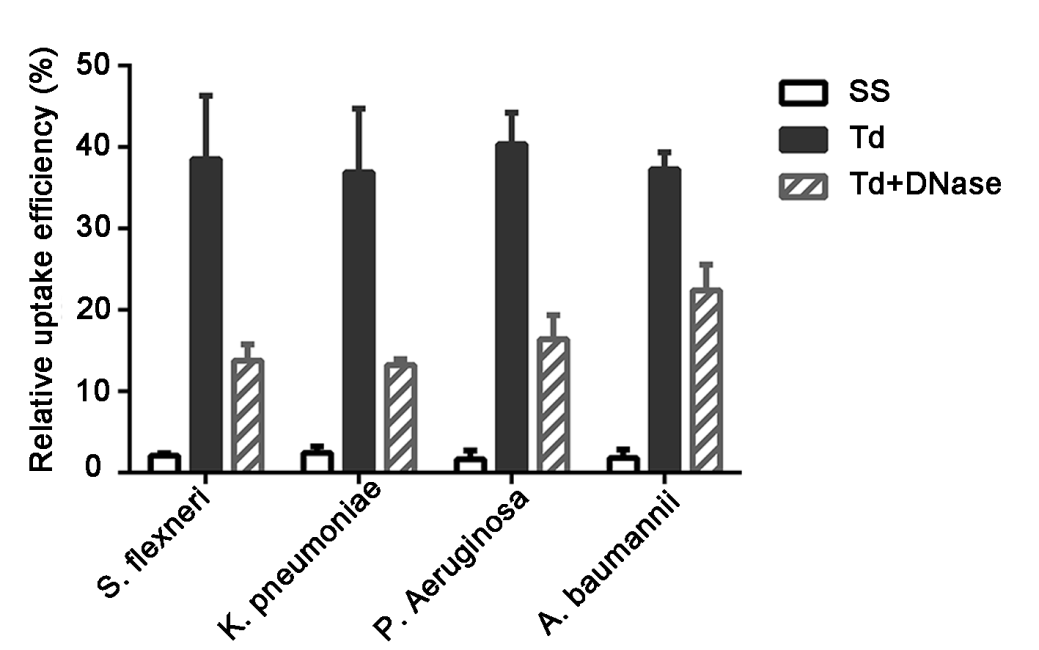
**

**Figure S3. Uptake efficiency of Td in different bacterial strains analyzed by flow cytometry.** The bacterial cells were incubated with Td (0.5 μM) for 1.5 h and then were either treated or not treated with DNase before flow cytometry analysis. SS: single-strand DNA; Td: DNA tetrahedron. SS was used as a control.


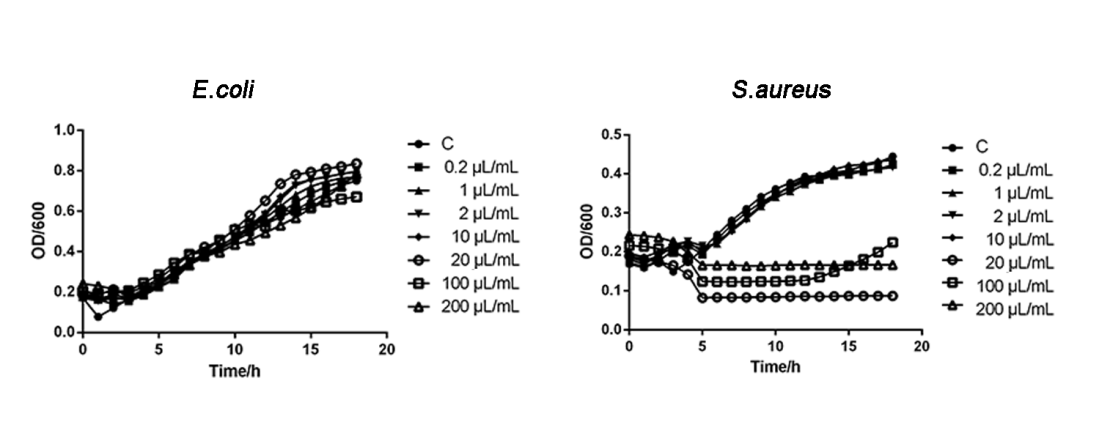


**Figure S4. Bacterial toxicity of LP2000.** Bacteria were treated with LP2000 at different concentrations, and a growth assay was conducted for 18 h.


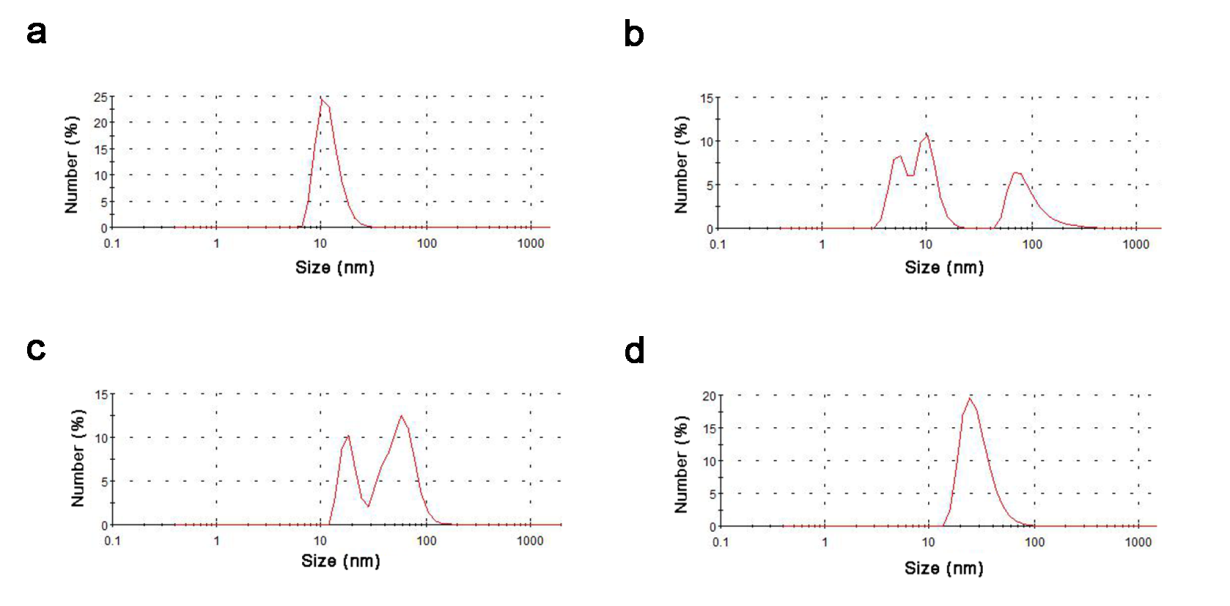


**Figure S5. Hydrodynamic size of Td mixed with LP2000 at various ratios of LP2000 (μL) to Td (μg) (LP2000/Td ratio) measured by DLS.** a) 0.0025 μL LP2000/μg Td. b) 0.0125 μL LP2000/μg Td. c) 0.025 LP2000 μL/μg Td. d) 0.125 μL LP2000/μg Td.

**
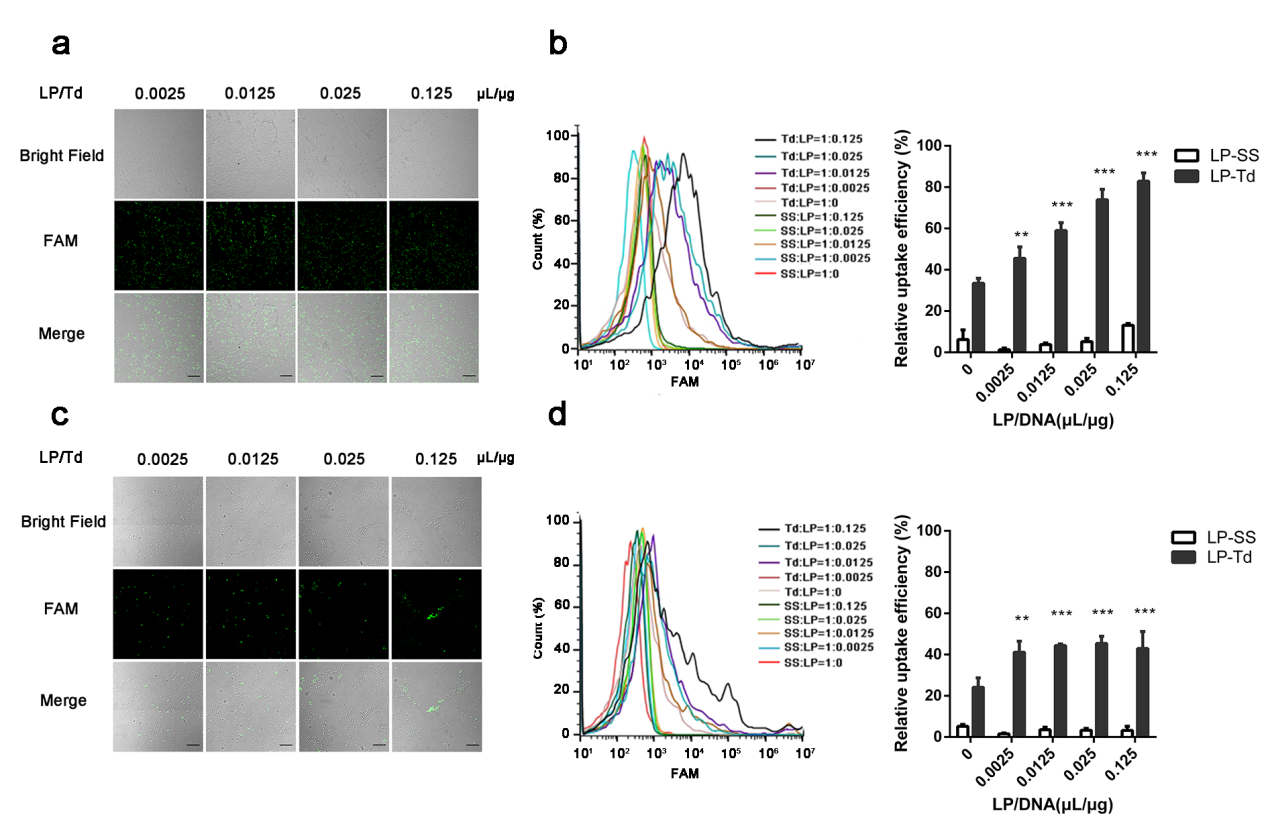
**

**Figure S6. Optimization of the uptake efficiency of Td by adding LP2000 at various ratios of LP2000 (μL) to Td (μg) (LP2000/Td ratio).** a) Confocal microscopy imaging and b) flow cytometry to analyze the uptake efficiency of Td at different LP2000/Td ratios (0.0025, 0.0125, 0.025, or 0.125 μL/μg) in *E. coli*. c) Confocal microscopy imaging and d) flow cytometry to analyze the uptake efficiency of Td at different LP2000/Td ratios (0.0025, 0.0125, 0.025, or 0.125 μL/μg) in *S. aureus*. SS: single-strand DNA; Td: DNA tetrahedron. SS was used as a control. “**”: *p*＜0.01; “***”: *p*＜0.001. P value versus the 0 μL/μg group.


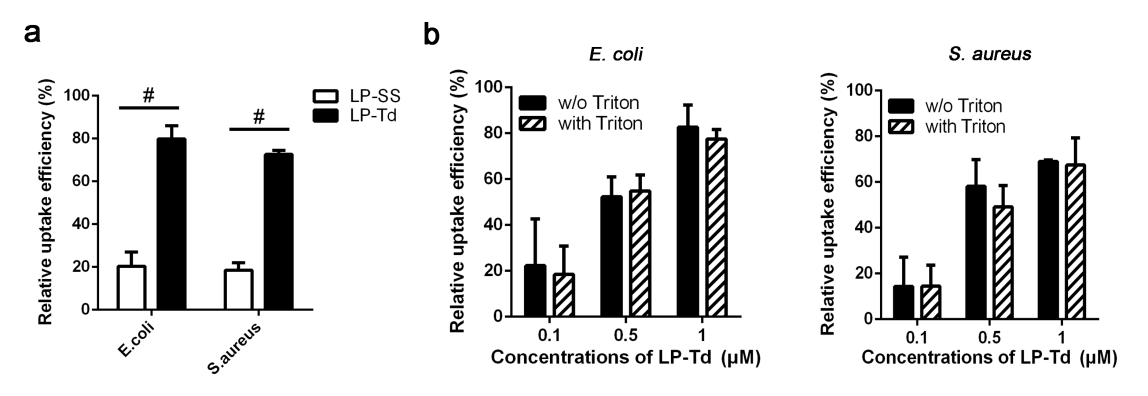


**Figure S7. Superiority of Td to cross bacterial membrane with the help of LP2000.** a) flow cytometry to analyze the uptake efficiency of single strand DNA or Td (1 μM) with the same absolute amount LP2000 ( LP2000/Td ratio was 0.125 μL/μg) in *E.coli* and *S. aureus.* b) flow cytometry to clarify the influence of Triton X-100 on bacterial uptake efficiency of LP-Td in *E.coli* and *S. aureus* . “#”: *p*＜0.0001


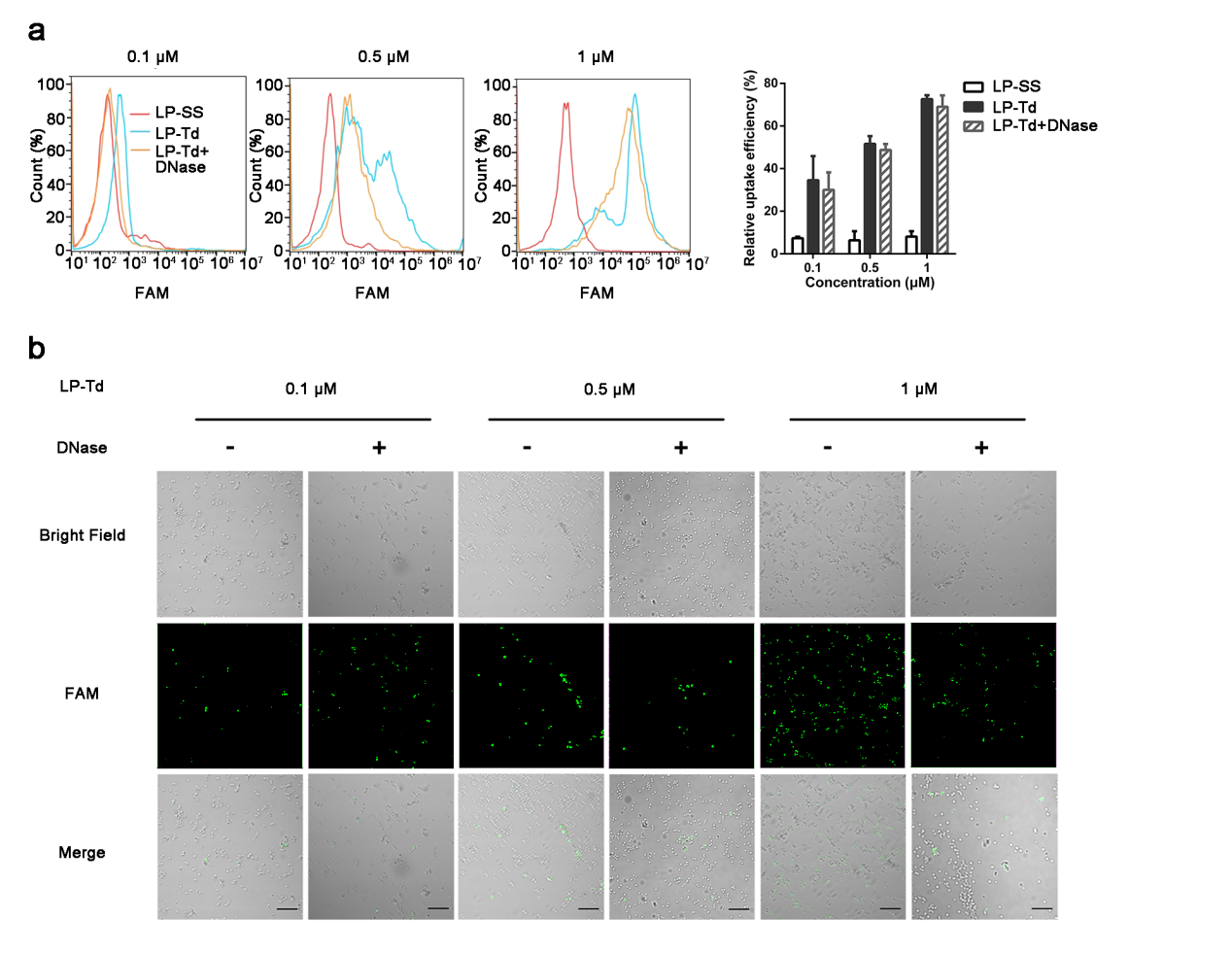


**Figure S8. Td uptake by *S.aureus* with the help of LP2000.** a) Flow cytometry and b) confocal microscopy imaging to analyze the uptake efficiency of Td mixed with LP2000 (LP-Td) by *S.aureus.* The bacterial cells were incubated with different concentrations of FAM-labeled LP-Td (0.1, 0.5, or 1 μM) for 1.5 h and then either treated or not treated with DNase before flow cytometry and confocal microscopy analyses. The LP2000/Td ratio was 0.125 μL/μg. SS: single-strand DNA; Td: DNA tetrahedron; LP: LP2000. SS was used as a control. Scale bars represent 10 μm.


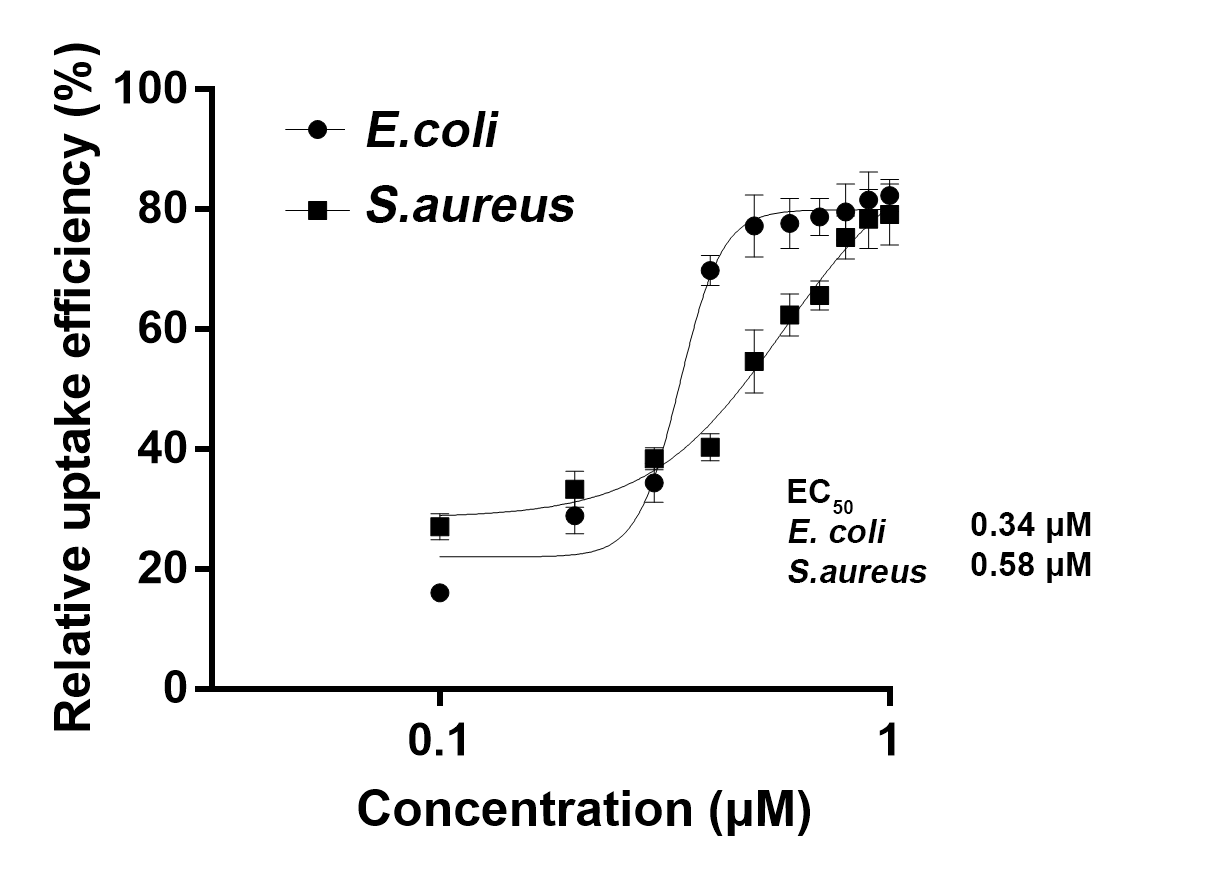


**Figure S9. The dose response curve for LP-Td uptake efficiency of *E. coli* and *S. aureus* respectively.**

**
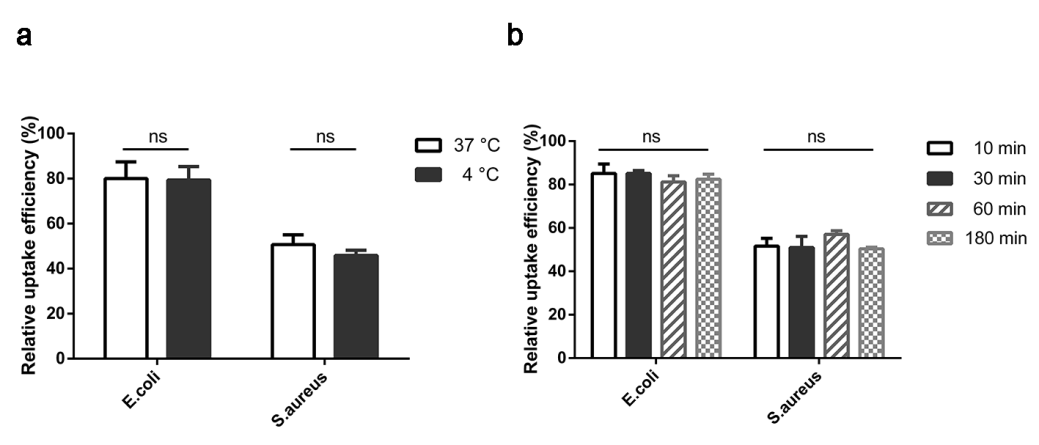
**

**Figure S10. The influence of temperature and time on bacterial uptake rates of LP-Td.** Bacteria were incubated with Td (0.5 μM) for 1.5 h. ns: no significance.


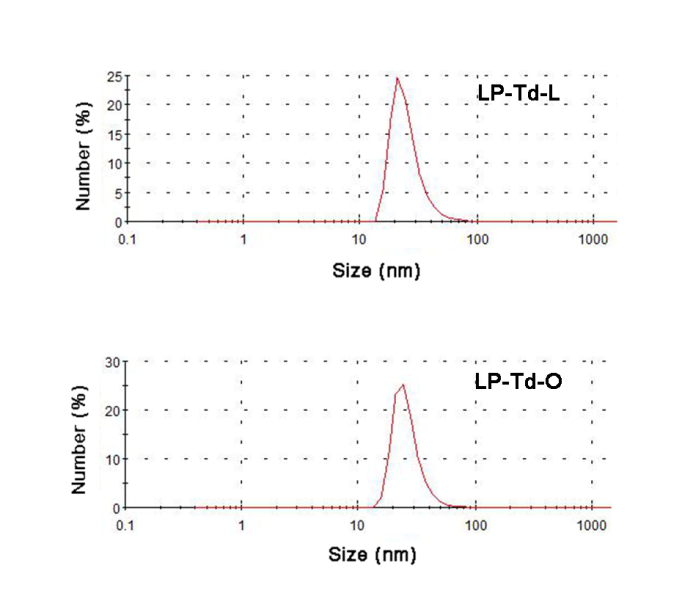


**Figure S11.** **Hydrodynamic size of LP-Td-O and LP-Tsd-L measured by DLS.** The LP2000/Td ratio was 0.125 μL/μg.
